# p63β modulates c-Myc activity via direct interaction and regulation of MM1 protein stability

**DOI:** 10.18632/oncotarget.10187

**Published:** 2016-06-20

**Authors:** Anning Han, Juan Li, Yimin Li, Yang Wang, Johann Bergholz, Yujun Zhang, Chenghua Li, Zhi-Xiong Xiao

**Affiliations:** ^1^ Center of Growth, Metabolism and Aging, Key Laboratory of Biological Resources and Ecological Environment of Ministry of Education, College of Life Sciences, Sichuan University, Chengdu 610064, China

**Keywords:** p63, MM1, c-Myc, protein stability, cell cycle

## Abstract

Both p53-related p63 and c-Myc are transcription factors playing key roles in cell proliferation, survival, development and tumorigenesis. In the present study, we identified that MM1, a c-Myc inhibitor, specifically binds to C-termini of p63β (including ΔNp63β and TAp63β). Further study demonstrates that p63β facilitates MM1 protein degradation via proteasomal pathway, resulting in elevation of c-Myc transactivation activity. Knockdown of ΔNp63β leads to decrease in c-Myc protein levels, concomitant with reduced expression of CDK4 and Cyclin D1, and impaired cell cycle progression, both of which are effectively reversed by simultaneous knockdown of MM1. Moreover, expression of p63 and CDK4 is concomitantly up-regulated in B-cell acute lymphoblastic leukemia. Together, this study reveals a novel crosstalk between p63 and c-Myc that may play an important role in cell cycle progression and tumorigenesis.

## INTRODUCTION

TP63, also known as the p63 gene, is a member of the p53 family, which locates on human chromosome 3q27–29 [1, 2]. Due to usage of alternative promoters, the p63 gene encodes two major classes of protein isoforms, TAp63 and ΔNp63. TAp63 proteins contain a full-length transactivation domain (TAD) at their N-termini that is homologous to that of p53, whereas ΔNp63 proteins possess incomplete N-terminal TADs. Owing to alternative splicing at the C-termini, TA or ΔN class of p63 can generate at least five different isoforms, including α, β, γ, δ, and ε [3]. p63α proteins, including TAp63α and ΔNp63α, contain two unique domains at the C-termini, namely, a sterile alpha motif (SAM) involved in protein-protein interaction and a transactivation-inhibitory domain (TID) [4]. However, each and every p63 isoform contains the same DNA binding domain (DBD) and oligomerization domain (OLD). It is known that ΔNp63α is the predominant isoform of p63 expressed in epithelial cells. Studies have shown that p63 proteins can function as transcription factors, controlling transcription of downstream genes, including p21, Bax, Puma, Dicer, MKP3, and genes involved in cell adhesion [5]. Consequently, p63 proteins play a key role in cell proliferation, survival, development and tumorigenesis [6].

c-Myc is an important transcription factor involved in cell proliferation, survival, differentiation and tumorigenesis. c-Myc modulates about 15% genes in organisms ranging from Drosophila to human, and is activated in about 20% malignant tumors [7, 8]. Downstream target genes of c-Myc include CDK4 [9, 10], Cyclin D1 [11], EZH2 [12], ECA39 [13, 14], eIF4E [15, 16], eIF-2α [15], and cdc25A [16].

MM1 (Myc modulator 1), also known as prefoldin subunit 5 (PFDN5), is one of six subunits of prefoldin, a molecular chaperone complex that binds and stabilizes newly synthesized polypeptides [17–20]. MM1 has been reported to bind to N-terminal transactivation domain of c-Myc (myc box 2) and represses c-Myc transactivity [17].

In this study, we report that p63α physically interacts with and destabilizes MM1, resulting in elevation of c-Myc transactivation activity.

## RESULTS

### MM1 binds to C-terminus of p63α

**Figure 1:**
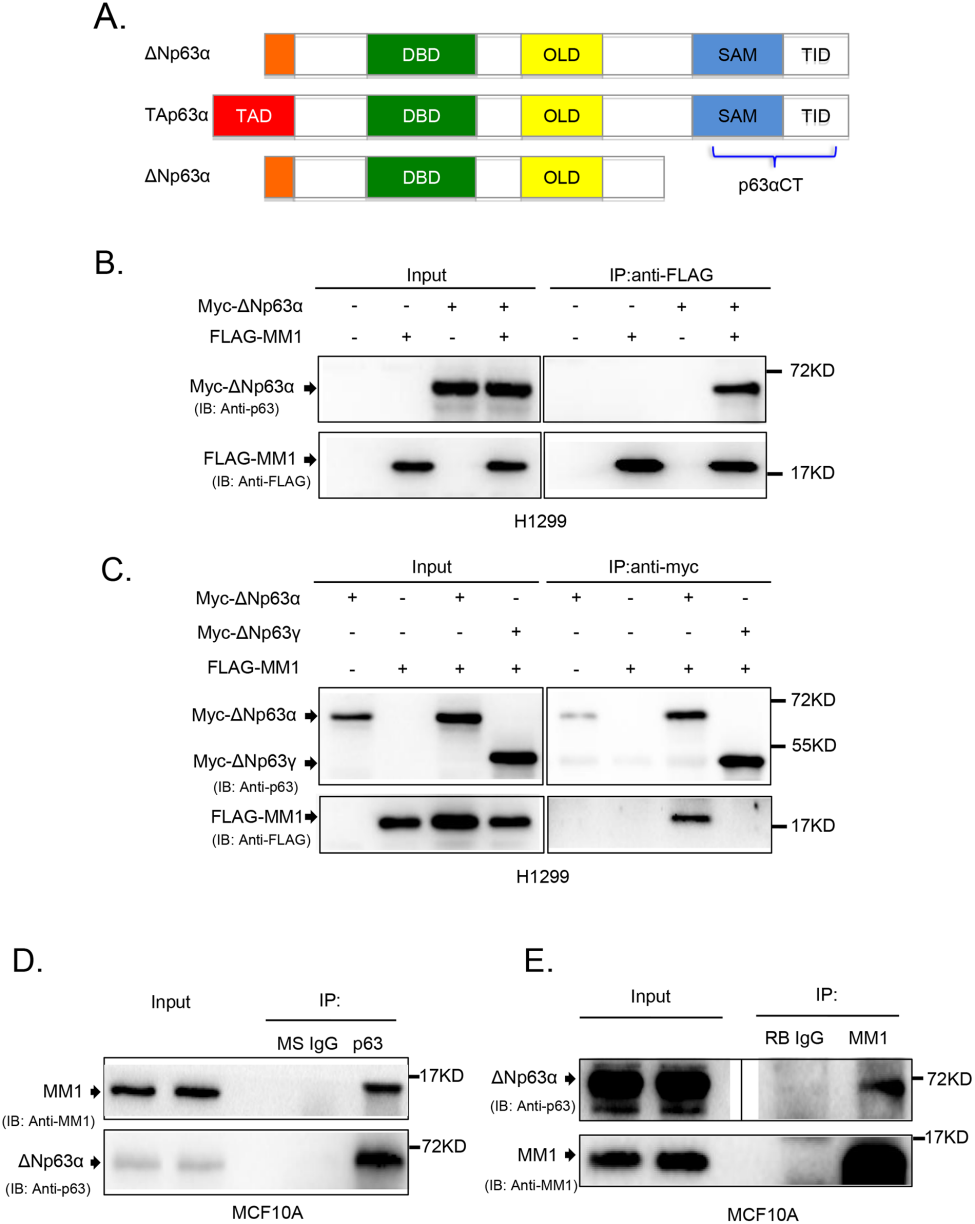
MM1 binds to C-terminus of p63α **A.** Schematic structure of p63 protein isoforms. TAD: transactivation domain; DBD: DNA-binding domain; OLD: oligomerization domain; SAM: sterile alpha motif; TID: transinhibitory domain. The C-terminal fragment of p63α (p63αCT), containing SAM and TID regions, was cloned as a “bait” for yeast two-hybrid screening. **B.** and **C.** ΔNp63α, but not ΔNp63γ, binds to MM1 in transfected cells. H1299 cells were co-transfected to express Myc-tagged ΔNp63α or ΔNp63γ plus FLAG-tagged MM1 proteins. Cell lysates were subjected to immunoprecipitation (IP) with antibody to FLAG or Myc tag. The immunoprecipitates were analyzed by immunoblotting (IB) with anti-p63 and anti-FLAG. Whole cell lysates were used as input controls. **D.** and **E.** Endogenous proteins of ΔNp63α and MM1 form stable complexes. MCF10A cell lysates were subjected to IP with anti-p63 or anti-MM1, using normal mouse IgG (MS IgG) or normal rabbit IgG (RB IgG), respectively, as a control. The immunoprecipitates were analyzed by IB with anti-p63 and anti-MM1. Whole cell lysates were used as input controls. Image of either short or long exposure was shown for ΔNp63α bands.

As shown in Figure 1A, p63α contains unique SAM and TID domains at the C-terminus (p63αCT), which are involved in protein-protein interaction and transactivation inhibition [21]. To identify proteins that interact with this region, we employed a yeast two-hybrid system to screen a human mammary gland cDNA library. As listed in Supplementary Table 2, 14 different proteins were found to associate with p63αCT, one of which was MM1. Since MM1 was previously reported to inhibit c-Myc, which is involved in cell cycle regulation, similar biological function that p63α possesses, we focused on MM1 and p63α in this study.

To confirm the interaction between MM1 and C-terminus of p63α in mammalian cells, we employed co-immunoprecipitation (co-IP) experiments using H1299 cells transiently co-overexpressing MM1 and different p63 isoforms. As shown in Figure 1B and 1C), ΔNp63α, but not ΔNp63γ, interacted with MM1. Further study revealed that endogenous ΔNp63α and MM1 proteins formed stable complexes in MCF10A cells (Figure 1D and 1E).

### Expression of p63α down-regulates MM1 protein levels

**Figure 2:**
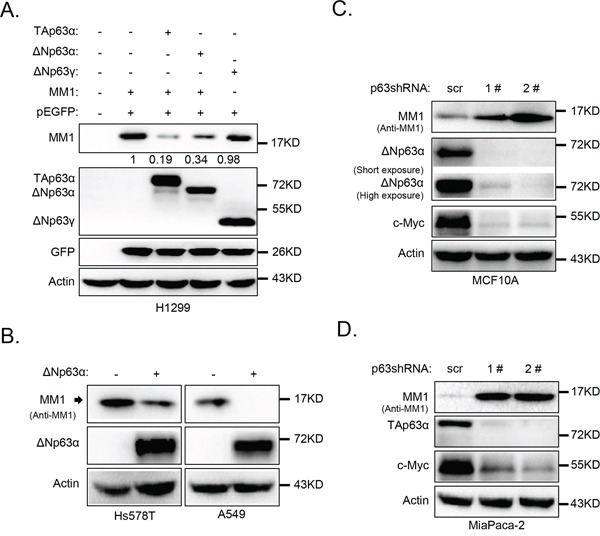
p63α down-regulates MM1 protein expression **A.** ΔNp63α down-regulates MM1 expression in H1299 cells. H1299 cells were transfected with indicated expression plasmids. 24 hours post transfection, cells were harvested and subjected to IB analysis for p63, MM1, GFP and Actin. Intensities of MM1 bands were normalized with GFP bands, and that in lane 2 was set as 1. **B.** ΔNp63α down-regulates MM1 in Hs578T and A549 cells. Hs578T or A549 cells were infected with lentiviral-based ΔNp63α. 48 hours post infection, cells were lysed and subjected to IB analysis for p63, MM1 and Actin. **C.** and **D.** Ablation of endogenous ΔNp63α or TAp63α up-regulates endogenous MM1. MCF10A or MiaPaCa-2 cells were infected with lentiviral-based shRNA 1# or 2#, specific for pan p63, using lentiviral-based scrambled shRNA as a control (scr). 48 hours post infection, cells were lysed and subjected to IB analysis for p63, c-Myc, MM1 and Actin.

We previously reported that peptidyl-prolyl isomerase Pin1 directly binds to and stabilizes p63α protein [22]. To investigate whether MM1 can influence ΔNp63α protein levels, we co-expressed MM1 and ΔNp63α in H1299 cells. As shown in Figure 2A, expression of either ΔNp63α or TAp63α significantly decreased protein level of MM1, but not GFP. By contrast, ΔNp63γ failed to affect MM1. Further investigation reveals that ectopic expression of ΔNp63α resulted in significant decrease of endogenous MM1 protein levels in Hs5787T and A549 cells (Figure 2B).

We next knocked down endogenous ΔNp63α in MCF10A cells, which are non-transformed human breast epithelial cells predominantly expressing ΔNp63α isoform, using lentiviral-based short hairpin RNAs (shRNAs). As shown in Figure 2C, two different shRNAs against pan p63 effectively knocked down endogenous ΔNp63α, resulting in significant increase of endogenous MM1. In addition, acute depletion of ΔNp63α led to decrease in c-Myc protein levels (Figure 2C), consistent with a previous report that p63 is necessary for proper expression of c-Myc via Wnt/β-catenin and Notch pathways in human keratinocytes [23].

To investigate whether TAp63α has the same effects to MM1 and c-Myc as that of ΔNp63α, we used a human pancreatic cancer cell line, MiaPaCa-2, which predominantly expresses TAp63α isoform [24]. As shown in Figure 2D, knockdown of TAp63α in MiaPaCa cells led to a significant increase in MM1 and decreases in c-Myc protein levels. These data indicate that both TAp63α and ΔNp63*α* isoforms can down-regulate MM1 protein expression.

### ΔNp63α promotes MM1 proteasome-dependent degradation

**Figure 3:**
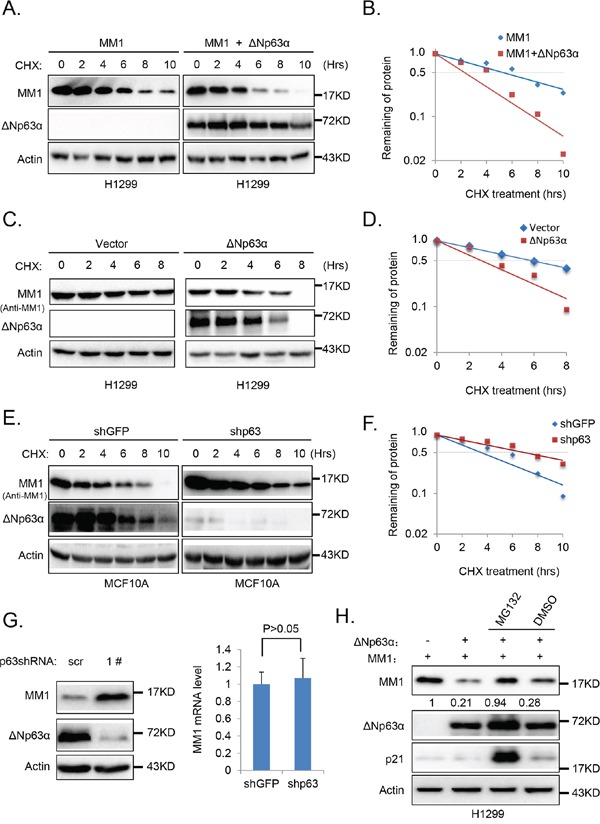
ΔNp63α promotes MM1 proteasome-dependent degradation **A.** Ectopic expression of ΔNp63α shortens half-life of MM1 protein. H1299 cells were co-transfected with FLAG-MM1 together with vector control (FLAG-MM1) or ΔNp63α (FLAG-MM1 + ΔNp63α). 24 hours post transfection, cells were treated with 100 μg/ml cycloheximide (CHX) for indicated time intervals. Cells were harvested and subjected to IB analysis. **B.** Quantification results of panel A. Percentages of FLAG-MM1 protein were normalized with Actin, and that for the time point of 0 hour was set as 100. **C.** and **D.** Ectopic expression of ΔNp63α shortens half-life of endogenous MM1 protein. H1299 cells transfected with vector control or ΔNp63α were subjected to measurement of MM1 protein half-life, using methods abovementioned. **E.** and **F.** Depletion of ΔNp63α extends half-life of endogenous MM1 protein. MCF10A cells were infected with lentiviral-based shRNA specific for p63 (1#) to knockdown endogenous ΔNp63α, using lentiviral-based shRNA specific for GFP as a control. 48 hours post infection, cells were lysed and subjected to measurement of MM1 protein half-life. **G.** Knockdown of endogenous p63 up-regulates endogenous MM1. MCF10A cells were infected with lentiviral-based scrambled or p63 (1#) shRNAs. 72 hours post infection, cells were subjected to IB or Q-PCR analysis. The Q-PCR data were presented as as means±*s.e.* to measure mRNA levels of MM1, from three independent experiments performed in triplicate. **H.** Inhibition of proteasome abrogates ΔNp63α-induced down-regulation of MM1. H1299 cells were co-transfected with MM1 plasmid plus vector control or ΔNp63α plasmid. 24 hours post transfection, cells were treated with MG132 or DMSO for additional 6 hours. Cells were then harvested and subjected to immunoblot analysis. Intensities of MM1 bands were normalized with Actin bands.

To examine whether ΔNp63α-mediated reduction of MM1 protein is due to altered protein stability, we examined the effects of ΔNp63α on co-expressed MM1 in H1299 cells. The MM1 protein half-life was measured with cycloheximide (CHX) chase assay. As shown in Figure 3A and 3B, the MM1 protein half-life was significantly shortened when co-expressed with ΔNp63α. Similarly, overexpression of ΔNp63α also decreased half-life of endogenous MM1 protein in H1299 cells (Figure 3C and 3D). Conversely, knockdown of endogenous ΔNp63α in MCF10A cells extended half-life of endogenous MM1 protein (Figure 3E and 3F). Furthermore, Q-PCR analyses indicated that MM1 mRNA levels were comparable in MCF10A cells with or without depletion of endogenous ΔNp63α (Figure 3G). These data demonstrate that ΔNp63α promotes MM1 protein degradation.

To determine whether ΔNp63α destabilizes MM1 through proteasome, we used MG132 to inhibit proteasome in H1299 cells co-transfected with MM1 and ΔNp63α. As shown in Figure 3H, p21^cip1^, which is mainly degraded through the proteasomal pathway [25], was dramatically increased by treatment with MG132, indicating the effectiveness of MG132; MG132 significantly blocked ΔNp63α-mediated MM1 protein degradation. These data suggest that ΔNp63α promotes proteasome-dependent degradation of MM1 protein.

### Ablation of ΔNp63α down regulates c-Myc transactivity and impair cell cycle progression

**Figure 4:**
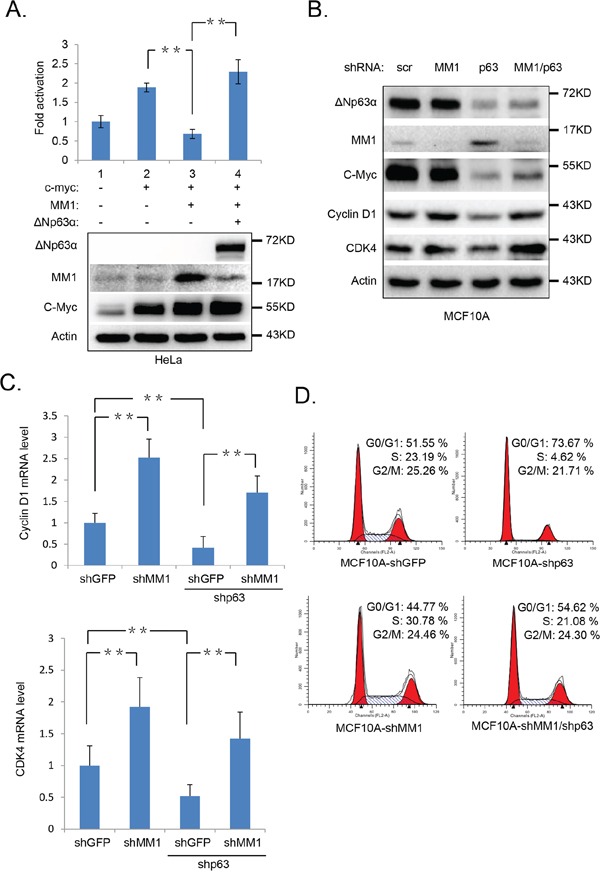
Ablation of ΔNp63α down regulates c-Myc transactivity and impair cell cycle progression **A.** ΔNp63α derepresses MM1-mediated suppression of c-Myc transactivity. HeLa cells were co-transfected with MBC1-4-Luc reporter and Renilla plus indicated plasmids. All transfections were performed in triplicates. 48 hours post transfection, cells were lysed and subjected to luciferase assay and IB analysis, respectively. The expression of MBC4-Luc reporter was normalized to Renilla luciferase activity. Results were presented as fold activation of luciferase activity with standard deviation (n=3). **, p<0.01. **B-D.** Knockdown of ΔNp63α down regulates c-Myc and its downstream targets, resulting in cell cycle arrest, which are rescued by shMM1. MCF10A cells were infected with lentiviral-based scrambled, MM1 or (and) p63 (1#) shRNAs. 72 hours post infection, cells were subjected to IB (B), Q-PCR (C), or FACS (D) analysis. The Q-PCR data were presented as means ± *s.e.* to measure mRNA levels of Cyclin D1 and CDK4, from three independent experiments performed in triplicate. **, p<0.01.

c-Myc is a transcription factor which recognizes and binds to the E-box sequences in gene promoters and activates their transcription [26–28]. It's reported that MM1 represses c-Myc-mediated E-box-dependent transcription [20]. To test whether ΔNp63α-mediated down-regulation of MM1 increases c-Myc-mediated transactivation, we performed a set of luciferase assays, using the MBC1-4-Luc reporter containing 4 E-box promoter regions of cyclin-dependent kinase 4 (CDK4) gene, which is a transcriptional target of c-Myc [9]. As shown in Figure 4A, c-Myc activated the luciferase expression of MBC1-4-Luc, which was suppressed by MM1, whereas ΔNp63α significantly rescues this MM1-mediated repression of MBC1-4-Luc. These data suggest that ΔNp63α stimulates c-Myc-mediated transactivation via down-regulating MM1.

To investigate the influence of endogenous ΔNp63α and MM1 on c-Myc transactivity, we knocked down p63α or (and) MM1 in MCF10A cells with lentiviral-based shRNAs. As shown in Figure 4B, knockdown of MM1 led to an increase in expression of Cyclin D1 and CDK4, which are both transcriptional target of c-Myc and are important for cell cycle G1 phase progression [9, 11]; depletion of ΔNp63α resulted in decreases of Cyclin D1 and CDK4 expressions, which were significantly reversed by simultaneous knockdown of MM1. Further study reveals that this regulation of CDK4 and Cyclin D1 expression mediated by p63 and MM1 occurs at mRNA levels (Figure 4C). These data suggest that endogenous p63α can positively elevate intrinsic c-Myc transactivity via inhibition of MM1. Additionally, simultaneous knockdown of MM1 could rescue, at least in part, down-regulation of c-Myc protein level induced by p63 ablation (Figure 4B). consistent with previous reports that p63 proteins are necessary for basal transcription of c-Myc gene [23], and MM1 may promote Cullin2-mediated degradation of c-Myc protein [29].

Both c-Myc and p63α are transcription factors involved in cell cycle progression [8, 30–32]. To investigate whether p63α-induced destabilization of MM1 can impact cell cycle, we performed a flow cytometry analysis with MCF10A cells infected with shMM1 or (and) shp63. As shown in Figure 4D, knockdown of p63α significantly decreased percentage of cells in S phase, while simultaneous knockdown of ΔNp63α and MM1 led to cell cycle distribution similar to that of control. This suggests that p63α can positively regulate cell cycle progression via inhibition of MM1.

### Correlation of elevation of p63 and c-Myc targets in tumorigenesis

**Figure 5:**
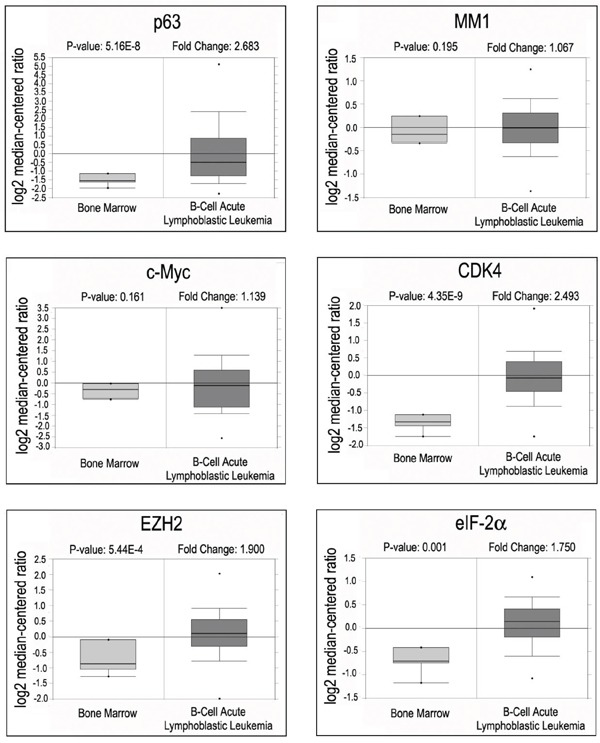
Oncomine analyses of p63, MM1, c-Myc and c-Myc target genes in B-cell acute lymphoblastic leukemia Box plots representing expression of p63, MM1, c-Myc and its responsive genes from B-cell acute lymphoblastic leukemia samples (Andersson data set).

To explore the clinical relevance of c-Myc activation mediated by p63α/MM1 pathway, we analyzed Oncomine, an online microarray database. As shown in Figure 5, mRNA levels of p63 and several transcriptional targets of c-Myc, including CDK4, EZH2 and eIF-2α, are concomitantly up-regulated in human B-cell acute lymphoblastic leukemia specimens, while mRNA levels of MM1 and c-Myc remain largely unchanged, suggesting that elevated p63α may stimulate c-Myc transactivity in some cancer types, such as B-cell acute lymphoblastic leukemia.

## DISCUSSION

p63 and p73 are both members of p53 family. It has been reported that c-Myc inhibitor MM1 binds to C-terminus of p73α and enhances p73α-dependent transcription [33]. Our present study shows that MM1 associates with C-terminus of p63α, which is homologous to that of p73α. We found that p63α physically interacts with MM1 protein and facilitates its proteasomal degradation. Overexpression of p63α effectively shortens half-life of MM1 protein, while knockdown of p63α extends it.

As important transcription factors involved in cell cycle control and tumorigenesis, p63 and c-Myc regulate transcription of their downstream target genes. Recently, it was reported that p63 is required for basal transcription of c-Myc in human keratinocytes [23]. Our data demonstrate that overexpression of ΔNp63α can significantly reverse MM1-mediated repression of c-Myc transactivation, consistent with the observation that p63α destabilizes MM1 protein. Furthermore, knockdown of ΔNp63α in MCF10A cells impairs cell cycle progress, in keeping with our previous report [22]. Importantly, simultaneous knockdown of ΔNp63α and MM1 effectively restores cell cycle arrest induced by knockdown of ΔNp63α, indicating that MM1 is an important regulator in p63α-mediated cell cycle control, likely though modulation of c-Myc activity and function. Moreover, results of clinical analysis indicate that the p63α/MM1 pathway may derepress c-Myc transactivity in B-cell acute lymphoblastic leukemia.

We speculate that p63α can positively affect c-Myc by two different pathways: p63 proteins are necessary for basal transcription of c-Myc in some cell types, possibly via Wnt/β-catenin and Notch signaling pathways [23]; on the other hand, p63α promotes proteasomal degradation of MM1 and consequently derepresses its suppression effects on c-Myc [17, 20, 29]. Taken together, this study reveals a novel way that p63 up-regulates c-Myc activity and function, which may attribute to various biological and pathophysiological processes such as cell cycle and tumorigenesis.

## MATERIALS AND METHODS

### Yeast two-hybrid screening

The cDNA encoding C-terminal region of p63α (amino acid residues 535–680, p63αCT) was cloned into pGBKT7 as a “bait” plasmid. The MATCHMAKER GAL4 Two-Hybrid System 3 (Clontech) and a cDNA library derived from human mammary gland in the pACT2 yeast expression vector (Clontech) were used to screen interacting proteins of p63αCT according to the manufacturer's instructions.

### Cell culture and drug treatment

H1299, HeLa, 293T, A549, and MiaPaCa-2 cells were maintained in Dulbecco's modified Eagle's medium (DMEM, Hyclone) supplemented with 10% fetal bovine serum (FBS, Hyclone) and 1% penicillin/streptomycin (Hyclone). For Hs578T cells, DMEM was supplemented with 10% FBS plus 10 mg/ml insulin (Sigma). MCF10A cells were maintained in DMEM/F12 media (Hyclone) supplemented with 20 ng/ml epidermal growth factor (Invitrogen), 100 ng/ml cholera toxin (Sigma,), 10 mg/ml insulin (Sigma), 500 ng/ml hydrocortisone (Sigma), 1% penicillin/streptomycin (Hyclone) sulfate and 5% FBS (Hyclone). All cells were cultured at 37°C in a humidified 5% CO_2_ incubator. MG132 and cycloheximide (CHX) were purchased from Sigma.

### Transfections and immunoblot (IB) analysis

H1299 or HeLa cells with 50% confluency in 6-well plates were co-transfected with 400 ng pCMV2-FLAG-MM1, 200 ng pEGFP and 400 ng pcDNA3.1/pcDNA3.1-ΔNp63α/pcDNA3.1-ΔNp63γ with 2.5 μl lipofectamine 2000 (Invitrogen). 24 hours later, cells were collected, washed with phosphate-buffered saline, and resuspended in EBC250 lysis buffer (50 mM Tris-HCl, pH8.0, 250 mM NaCl, 0.5% Nonidet P-40, 0.2 mM phenylmethylsulfonyl fluoride, 2 μg/ml leupeptin, 2 μg/ml aprotinin, 50 mM NaF, and 0.5 mM Na_3_VO_4_). Protein concentration was determined using the Bio-Rad protein assay reagent (Bio-Rad). An equal amount of protein (about 50 μg total protein) was loaded, separated on a 10% SDS-PAGE, transferred to polyvinylidene difluoride membrane (Bio-Rad), and hybridized to an appropriate primary antibody and horseradish peroxidase-conjugated secondary antibody for subsequent detection with ECL (Millipore). IB analysis was performed with anti-p63 (Santa Cruz, sc-8431, 1:200), anti-FLAG (Sigma, F1804, 1:1000), anti-MM1 (Epitomics, 5689-1, 1:500), anti-c-Myc (Epitomics, 1472-1, 1:1000), anti-GFP (Santa Cruz, sc-9996, 1:1000), anti-p21 (Abcam, ab54562, 1:500), anti-Cyclin D1 (Epitomics, 2261-1, 1:1000), anti-CDK4 (Santa Cruz, sc-260, 1:1000), or anti-Actin (Abcam, ab13772, 1:1000).

### Co-immunoprecipitation (Co-IP)

H1299 cells co-transfected with indicated plasmids or MCF10A cells were lysed in ice-cold EBC250 lysis buffer for 30 min on ice. Lysates were cleared by centrifugation at 16,000 g for 15 min. Protein concentration was determined using the Bio-Rad protein assay reagent (Bio-Rad). Cell lysates containing 2 mg total protein were incubated with 2 μg anti-FLAG (Sigma, F1804), Myc tag antibody (Abcam, ab32), p63 antibody (Santa Cruz, sc-8431), or anti-MM1 (Epitomics, 5689-1), respectively, at 4°C for 2 hours. Then the immune complexes were precipitated with protein A-agarose at 4°C for 3 hours The immunoprecipitates were washed with EBC250 lysis buffer, separated by SDS-PAGE, and subjected to IB analysis with indicated antibodies.

### Quantitative PCR (Q-PCR) analysis

Total RNA was isolated using RNeasy Mini Kit (Qiagen), followed by reverse transcription using One-Step RT-PCR kit (Qiagen) according to themanufacturers’ instruction. Q-PCR was performed using QuantiTect SYBR Green PCR Kit (Qiagen) according to the manufacturer's instructions. Primer sequences were described in Supplementary Table 1. All primers spanned at least one intron and control amplification was performed on RNA samples not subjected to the reverse transcription, in parallel, to ensure no contaminating genomic DNA was present.

### Lentiviral infection

293T cells were cotransfected with pLVX-ΔNp63α/pLKO.1-p63shRNA/pLKO.1-MM1shRNA along with psPAX2 and pMD2.G lentiviral packaging plasmids by Lipofectamine 2000. The medium were collected and filtered through a 0.45 μM filter to remove debris 48 hours later. The lentiviral particles were then concentrated by ultra-centrifugation (20,000 rpm, 2h at 4°C), resuspended in fresh medium at 4°C for 24 h, then supplemented with polybrene (10μg/ml) and used to infect A549, Hs578T, MCF10A, or MiaPaCa-2 cells. 48 hours after infection, the cells were subjected to IB analysis or selected in growth medium supplemented with 2 μg/ml puromycin for two days.

### Protein stability assay

Cells were transiently transfected (or infected) with indicated plasmids or shRNA lentiviruses. 24 hours post transfection (or infection), cells were treated with Cycloheximide (CHX) at a final concentration of 100 μg/ml for 0~10 more hours. Cells were collected at the indicated time points. Equal amounts of total proteins were subjected to IB analysis. Intensities of bands for MM1 or Actin were quantified with Image Lab (Bio-Rad). The protein levels of MM1 were normalized with Actin, and that of the time point of 0 hour was set as 100 percent.

### Assays for proteasome-dependent MM1 Protein degradation

H1299 cells with 50% confluency in 6-well plates were co-transfected with 800 ng pCMV2-Flag-MM1 and 800 ng pcDNA3.1 or pcDNA3.1-ΔNp63α by lipofectamine 2000. 24 hours later, cells were treated with 20 μM MG132 or equal amount of solvent vehicle DMSO for 6 more hours. Then cells were collected and lysed with EBC250. Equal amounts of total protein were subjected to western for p63, p21, MM1, and Actin.

### Luciferase assays

HeLa cells were plated for transfection at a density of 2×10^5^ cells/well in 12-well plates for 24 hour. Cells were co-transfected with 100 ng MBC1-4-Luc reporter and 5 ng pRL-TK Renilla plasmids, plus c-Myc and (or) FLAG-MM1, ΔNp63α expression plasmids (200 ng each). The total amount of DNA was kept constant (705 ng per transfection) with pcDNA3.1 vector. Cells were harvested at 48 hours after transfection and lysed in Passive Lysis Buffer (Promega). Lysates were analyzed for firefly and Renilla luciferase activities using the Dual Luciferase Reagent Assay Kit (Promega). Luciferase assays were performed as described (Luciferase assay system; Promega). Luminescence was measured in a luminometer. Relative luciferase activity was determined by normalizing luciferase activity with Renilla.

### Flow cytometry (FACS) analysis

Cells were fixed in 70% ethanol at 4°C overnight and stained with 50 μg/ml propidium iodide (Sigma) supplemented with 100 μg/ml RNase A (Sigma) for 40 min at 37°C in the dark. Then cells were subjected to FCM analysis by FACScan flow cytometer (Becton Dickson). Data were analyzed using the Cell Quest program.

### Bioinformatic and statistical analysis

Oncomine (Compendia Bioscience, Ann Arbor, MI, USA) was used for bioinformatic analysis of gene expression. Quantitative data were analyzed statistically using Student's *t*-test to assess significance. Data are presented as means ± *s.e.*, as noted in figure legends.

## SUPPLEMENTARY TABLES



## References

[1] Yang A, Schweitzer R, Sun D, Kaghad M, Walker N, Bronson RT, Tabin C, Sharpe A, Caput D, Crum C, McKeon F (1999). p63 is essential for regenerative proliferation in limb, craniofacial and epithelial development. Nature.

[2] Yang A, Kaghad M, Wang Y, Gillett E, Fleming MD, Dotsch V, Andrews NC, Caput D, McKeon F (1998). p63, a p53 homolog at 3q27-29, encodes multiple products with transactivating, death-inducing, and dominant-negative activities. Mol Cell.

[3] Mangiulli M, Valletti A, Caratozzolo MF, Tullo A, Sbisa E, Pesole G, D'Erchia AM (2009). Identification and functional characterization of two new transcriptional variants of the human p63 gene. Nucleic Acids Res.

[4] Thanos CD, Bowie JU (1999). p53 Family members p63 and p73 are SAM domain-containing proteins. Protein Sci.

[5] Li C, Xiao ZX (2014). Regulation of p63 protein stability via ubiquitin-proteasome pathway. Biomed Res Int.

[6] Perez CA, Pietenpol JA (2007). Transcriptional programs regulated by p63 in normal epithelium and tumors. Cell Cycle.

[7] Nesbit CE, Tersak JM, Prochownik EV (1999). MYC oncogenes and human neoplastic disease. Oncogene.

[8] Dang CV (1999). c-Myc target genes involved in cell growth, apoptosis, and metabolism. Mol Cell Biol.

[9] Hermeking H, Rago C, Schuhmacher M, Li Q, Barrett JF, Obaya AJ, O'Connell BC, Mateyak MK, Tam W, Kohlhuber F, Dang CV, Sedivy JM, Eick D, Vogelstein B, Kinzler KW (2000). Identification of CDK4 as a target of c-MYC. Proc Natl Acad Sci U S A.

[10] Miliani de Marval PL, Macias E, Rounbehler R, Sicinski P, Kiyokawa H, Johnson DG, Conti CJ, Rodriguez-Puebla ML (2004). Lack of cyclin-dependent kinase 4 inhibits c-myc tumorigenic activities in epithelial tissues. Mol Cell Biol.

[11] Yang H, Li TW, Ko KS, Xia M, Lu SC (2009). Switch from Mnt-Max to Myc-Max induces p53 and cyclin D1 expression and apoptosis during cholestasis in mouse and human hepatocytes. Hepatology.

[12] Koh CM, Iwata T, Zheng Q, Bethel C, Yegnasubramanian S, De Marzo AM (2011). Myc enforces overexpression of EZH2 in early prostatic neoplasia via transcriptional and post-transcriptional mechanisms. Oncotarget.

[13] Benvenisty N, Leder A, Kuo A, Leder P (1992). An embryonically expressed gene is a target for c-Myc regulation via the c-Myc-binding sequence. Genes Dev.

[14] Schuldiner O, Eden A, Ben-Yosef T, Yanuka O, Simchen G, Benvenisty N (1996). ECA39, a conserved gene regulated by c-Myc in mice, is involved in G1/S cell cycle regulation in yeast. Proc Natl Acad Sci U S A.

[15] Rosenwald IB, Rhoads DB, Callanan LD, Isselbacher KJ, Schmidt EV (1993). Increased expression of eukaryotic translation initiation factors eIF-4E and eIF-2 alpha in response to growth induction by c-myc. Proc Natl Acad Sci U S A.

[16] Galaktionov K, Chen X, Beach D (1996). Cdc25 cell-cycle phosphatase as a target of c-myc. Nature.

[17] Mori K, Maeda Y, Kitaura H, Taira T, Iguchi-Ariga SM, Ariga H (1998). MM-1, a novel c-Myc-associating protein that represses transcriptional activity of c-Myc. J Biol Chem.

[18] Vainberg IE, Lewis SA, Rommelaere H, Ampe C, Vandekerckhove J, Klein HL, Cowan NJ (1998). Prefoldin, a chaperone that delivers unfolded proteins to cytosolic chaperonin. Cell.

[19] Sakamuro D, Prendergast GC (1999). New Myc-interacting proteins: a second Myc network emerges. Oncogene.

[20] Fujioka Y, Taira T, Maeda Y, Tanaka S, Nishihara H, Iguchi-Ariga SM, Nagashima K, Ariga H (2001). MM-1, a c-Myc-binding protein, is a candidate for a tumor suppressor in leukemia/lymphoma and tongue cancer. J Biol Chem.

[21] Straub WE, Weber TA, Schafer B, Candi E, Durst F, Ou HD, Rajalingam K, Melino G, Dotsch V (2010). The C-terminus of p63 contains multiple regulatory elements with different functions. Cell Death Dis.

[22] Li C, Chang DL, Yang Z, Qi J, Liu R, He H, Li D, Xiao ZX (2013). Pin1 modulates p63alpha protein stability in regulation of cell survival, proliferation and tumor formation. Cell Death Dis.

[23] Wu N, Rollin J, Masse I, Lamartine J, Gidrol X (2012). p63 regulates human keratinocyte proliferation via MYC-regulated gene network and differentiation commitment through cell adhesion-related gene network. J Biol Chem.

[24] Xu E, Zhang J, Zhang M, Jiang Y, Cho SJ, Chen X (2014). RNA-binding protein RBM24 regulates p63 expression via mRNA stability. Mol Cancer Res.

[25] Kibbe MR, Nie S, Seol DW, Kovesdi I, Lizonova A, Makaroun M, Billiar TR, Tzeng E (2000). Nitric oxide prevents p21 degradation with the ubiquitin-proteasome pathway in vascular smooth muscle cells. J Vasc Surg.

[26] Luscher B, Eisenman RN (1990). New light on Myc and Myb. Part I. Myc. Genes Dev.

[27] Blackwood EM, Eisenman RN (1991). Max: a helix-loop-helix zipper protein that forms a sequence-specific DNA-binding complex with Myc. Science.

[28] Blackwood EM, Luscher B, Eisenman RN (1992). Myc and Max associate in vivo. Genes Dev.

[29] Kimura Y, Nagao A, Fujioka Y, Satou A, Taira T, Iguchi-Ariga SM, Ariga H (2007). MM-1 facilitates degradation of c-Myc by recruiting proteasome and a novel ubiquitin E3 ligase. Int J Oncol.

[30] Dohn M, Zhang S, Chen X (2001). p63alpha and DeltaNp63alpha can induce cell cycle arrest and apoptosis and differentially regulate p53 target genes. Oncogene.

[31] Dang CV (2012). MYC on the path to cancer. Cell.

[32] Dang CV, O'Donnell KA, Zeller KI, Nguyen T, Osthus RC, Li F (2006). The c-Myc target gene network. Semin Cancer Biol.

[33] Watanabe K, Ozaki T, Nakagawa T, Miyazaki K, Takahashi M, Hosoda M, Hayashi S, Todo S, Nakagawara A (2002). Physical interaction of p73 with c-Myc and MM1, a c-Myc-binding protein, and modulation of the p73 function. J Biol Chem.

