# Correction: p63α modulates c-Myc activity via direct interaction and regulation of MM1 protein stability

**DOI:** 10.18632/oncotarget.18439

**Published:** 2017-06-12

**Authors:** Anning Han, Juan Li, Yimin Li, Yang Wang, Johann Bergholz, Yujun Zhang, Chenghua Li, Zhi-Xiong Xiao

**Present:** In the title and abstract, the ‘β’ symbol was mistakenly inserted instead of ‘α’.

**p63β modulates c-Myc activity via direct interaction and regulation of MM1 protein stability**

Anning Han^1^, Juan Li^1,*^, Yimin Li^1,*^, Yang Wang^1^, Johann Bergholz^1^, Yujun Zhang^1^, Chenghua Li^1^ and Zhi-Xiong Xiao^1^

This article corrects: p63β modulates c-Myc activity via direct interaction and regulation of MM1 protein stability, Volume 7, Issue 28, 44277–44287. Article first published online: 2016 Jun 20.

Link of original article: https://www.ncbi.nlm.nih.gov/pmc/articles/PMC5190095/

doi: 10.18632/oncotarget.10187

**Correct:** The proper title and abstract appear below.

The title should read

**p63α modulates c-Myc activity via direct interaction and regulation of MM1 protein stability**

All symbols of “β” in the ABSTRACT should be changed into “α”.

**ABSTRACT**

Both p53-related p63 and c-Myc are transcription factors playing key roles in cell proliferation, survival, development and tumorigenesis. In the present study, we identified that MM1, a c-Myc inhibitor, specifically binds to C-termini of p63α (including ΔNp63α and TAp63α). Further study demonstrates that p63α facilitates MM1 protein degradation via proteasomal pathway, resulting in elevation of c-Myc transactivation activity. Knockdown of ΔNp63α leads to decrease in c-Myc protein levels, concomitant with reduced expression of CDK4 and Cyclin D1, and impaired cell cycle progression, both of which are effectively reversed by simultaneous knockdown of MM1. Moreover, expression of p63 and CDK4 is concomitantly up-regulated in B-cell acute lymphoblastic leukemia. Together, this study reveals a novel crosstalk between p63 and c-Myc that may play an important role in cell cycle progression and tumorigenesis.

Original article: Oncotarget. 2016; 7:44277-44287. doi: 10.18632/oncotarget.10187

