# Supplementary material for: p63β modulates c-Myc activity via direct interaction and regulation of MM1 protein stability
Source: Oncotarget. 2016 Jun 20;7(28):44277–87. doi: 10.18632/oncotarget.10187 (PMC5190095; doi:10.18632/oncotarget.10187)
Supplement: Supplementary file 1 [file oncotarget-07-44277-s001.pdf]

p63α modulates c-Myc activity via direct interaction and regulation of MM1 protein stability

SUPPLEMENTARY TABLES

Supplementary Table S1: Primers for Q-PCR

Gene	Sequence (from 5' to 3')
MM1	Forward: GAACAAGAGCAACGAGGGGA<br>Reverse: AAGTCCTTGGCATCCTCAGC
CDK4	Forward: CAGATGGCACTTACACCCGT<br>Reverse: CAACTGGTCGGCTTCAGAGT
Cyclin D1	Forward: CAAGGCCTGAACCTGAGGAG<br>Reverse: CTTGGGGTCCATGTTCTGCT

Supplementary Table S2: List of clones encoding p63αCT-associated peptides according to yeast two hybrid

No.	Gene name	Full name
A5	IGH	immunoglobulin heavy locus
A10	EIF1	eukaryotic translation initiation factor 1
B29	EEF1A1	eukaryotic translation elongation factor 1 alpha 1
A13b	RARRES2	retinoic acid receptor responder (tazaroene induced) 2
A25	PHF12	PHD finger protein 12
B11	PDXK	pyridoxal (pyridoxine, vitamin B6) kinase
B24	GNB2L1	guanine nucleotide binding protein (G protein), betapolypeptide 2-like 1
B28	UBA52	ubiquitin A-52 residue ribosomal protein fusion product 1
C16	PFDN5/MM1	prefoldin subunit 5 isoform alpha, or Myc modulator 1
C24	PROS1	protein S (alpha)
C27	ADAMTS2	ADAM metallopeptidase with thrombospondin type 1 motif
D27	FBLN5	fibulin 5
C30	ALDOA	aldolase A, fructose-bisphosphate
D32	KIAA1731	KIAA1731
